# Highly efficient conversion of plant oil to bio-aviation fuel and valuable chemicals by combination of enzymatic transesterification, olefin cross-metathesis, and hydrotreating

**DOI:** 10.1186/s13068-018-1020-4

**Published:** 2018-02-07

**Authors:** Meng Wang, Mojin Chen, Yunming Fang, Tianwei Tan

**Affiliations:** 1 National Energy Research Center for Biorefinery, College of Life Science and Technology, Beijing University of Chemical Technology, Beijing, 100029 People’s Republic of China; 2 Department of Chemical Engineering, Beijing University of Chemical Technology, Beijing, 100029 People’s Republic of China

**Keywords:** Plant oil, *Candida* sp. 99–125 lipase, Olefin cross-metathesis, Bio-aviation fuel, α-olefin

## Abstract

**Background:**

The production of fuels and chemicals from renewable resources is increasingly important due to the environmental concern and depletion of fossil fuel. Despite the fast technical development in the production of aviation fuels, there are still several shortcomings such as a high cost of raw materials, a low yield of aviation fuels, and poor process techno-economic consideration. In recent years, olefin metathesis has become a powerful and versatile tool for generating new carbon–carbon bonds. The cross-metathesis reaction, one kind of metathesis reaction, has a high potential to efficiently convert plant oil into valuable chemicals, such as α-olefin and bio-aviation fuel by combining with a hydrotreatment process.

**Results:**

In this research, an efficient, four-step conversion of plant oil into bio-aviation fuel and valuable chemicals was developed by the combination of enzymatic transesterification, olefin cross-metathesis, and hydrotreating. Firstly, plant oil including oil with poor properties was esterified to fatty acid methyl esters by an enzyme-catalyzed process. Secondly, the fatty acid methyl esters were partially hydrotreated catalytically to transform poly-unsaturated fatty acid such as linoleic acid into oleic acid. The olefin cross-metathesis then transformed the oleic acid methyl ester (OAME) into 1-decene and 1-decenoic acid methyl ester (DAME). The catalysts used in this process were prepared/selected in function of the catalytic reaction and the reaction conditions were optimized. The carbon efficiency analysis of the new process illustrated that it was more economically feasible than the traditional hydrotreatment process.

**Conclusions:**

A highly efficient conversion process of plant oil into bio-aviation fuel and valuable chemicals by the combination of enzymatic transesterification, olefin cross-metathesis, and hydrotreatment with prepared and selected catalysts was designed. The reaction conditions were optimized. Plant oil was transformed into bio-aviation fuel and a high value α-olefin product with high carbon utilization.

**Electronic supplementary material:**

The online version of this article (10.1186/s13068-018-1020-4) contains supplementary material, which is available to authorized users.

## Background

Due to the increasing environmental concern and shortening of fossil fuel resources, the production of alternative fuels from renewable resources has fostered research and industrial activities [1–4]. A variety of technologies have been developed to produce renewable fuels, such as biodiesel, bio-ethanol, bio-butanol, and bio-aviation fuel from biomass feedstocks [5–7]. Advances in genetics, biotechnology, process chemistry, and engineering are leading to a new manufacturing concept for converting renewable biomass to valuable fuels and products, generally referred to as the biorefinery concept [5]. Among these renewable fuels, bio-aviation fuel, a complex hydrocarbon mixtures consisting of different classes, such as paraffin (C8–C15), naphthene, and aromatics [8, 9], is highly valuable with also higher requirements on low-temperature properties and energy density. The production of bio-aviation fuel from biomass feedstock has become a hot research topic in recent years, e.g., UOP/Honeywell developed the hydrogenated esters and fatty acids (HEFA) processes to upgrade vegetable oils and fats to renewable jet/diesel fuels [10–12].

Despite the fast technical development in the production of aviation fuels, there are still some shortcomings such as a high cost of raw materials and a low yield of aviation fuels [6]. The use of inexpensive vegetable oils, fats as feedstock and the development of new processes with high yield for bio-aviation fuel production are effective ways to reduce the costs [13]. Olefin metathesis has become a powerful and versatile tool for generating new carbon–carbon bonds [14–16] with ruthenium-based homogeneous catalysts that are tolerant to a broad range of functional groups, such as alcohols, amides, aldehydes, and carboxylic acids [17, 18]. The cross-metathesis (CM) reaction, one kind of metathesis reaction, enables to efficiently convert plant oil into valuable chemicals, such as α-olefin, and bio-aviation fuel by combination with a hydrotreatment process.

Catalytic hydrogenation is widely used in the current petrochemical industry [19]. Veldsink et al. presented a comprehensive literature review of heterogeneous hydrogenation of vegetable oils in 1997 [20]. In traditional hydrocarbon fuel objected hydrogenation processes, sulfide Co–Mo and Ni–Mo catalysts are generally used to efficiently remove S, N, and O from oil under high temperature and high pressure. However, such harsh hydrotreating conditions are inappropriate for oxygen-rich bio-oxygenate hydrodeoxygenation [3]. Recent studies showed that noble metal-based catalysts could show a high hydrodeoxygenation activity at lower temperatures [21, 22]. Further studies found that physical and chemical properties of support materials play important roles in the catalytic performance. Alumina, silica, active carbon, and zeolites have been investigated as the supports of catalysts [23–26]. The results proved that the acidities and pore structures importantly influence the catalytic activity.

Based on the above discussion, cross-metathesis may open up a new route for valuable chemicals and yields high bio-aviation fuel production especially when integrating with catalytic hydrotreating. However, metathesis reaction calls for high purity of the reactants. The purity of the feed stream usually has direct effect on reaction efficiency in metathesis reaction [27]. For this reason, some poor-quality lipid, such as waste cooking oil and crude plant oil, cannot be directly used as raw materials in metathesis reaction system without pre-treatment. Therefore, an efficient, environmental friendly and widely applicable pre-treatment process was needed for the application of poor-quality lipid in metathesis reaction. Enzyme-catalyzed esterification/transesterification between lipid and alcohol, which is environmental friendly and widely applicable [28], is an ideal pre-treatment process for using poor-quality lipid in metathesis reaction.

**Scheme 1:**
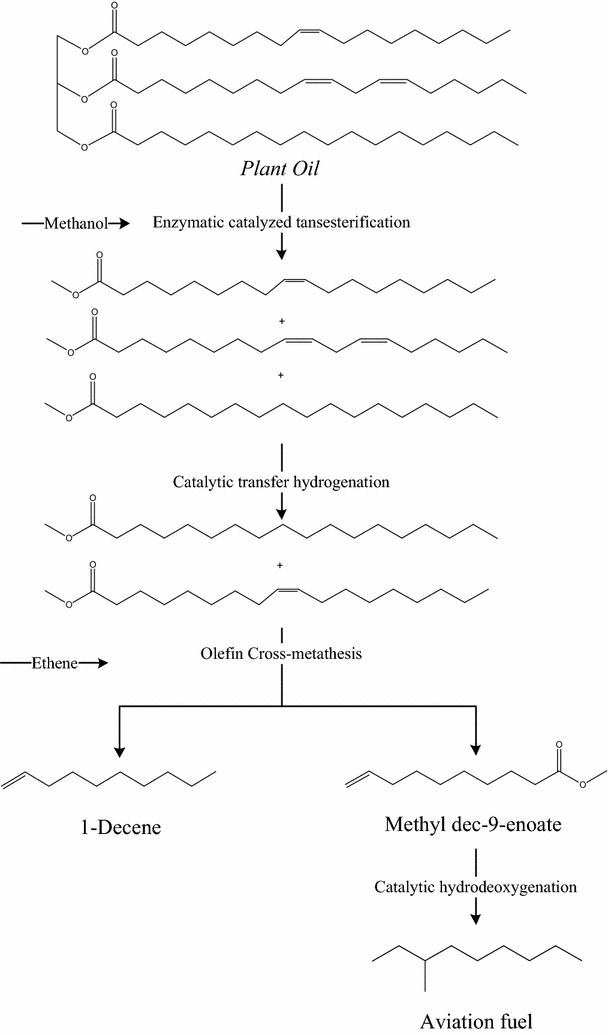
The designed process for jet fuel and α-olefin production from plant oil

Hence in this paper, a novel process shown in Scheme 1, which allows all carbons in the fatty acid chain of plant oil be converted into high valuable chemicals and bio-aviation fuel with commercially available and state-of-the-art catalysts was developed. Details of catalysts selection and development, including process conditions screening for such process were discussed. Furthermore, the economic assessment of the developed process was also carried out for the comparison with traditional processes.

## Results and discussion

### Process introduction

As the use of inexpensive plant oil as feedstock was an efficient way to reduce the cost of bio-aviation fuel and valuable chemicals’ production [13], a non-edible plant oil, crude *Amygdalus Pedunculata* plant oil was used as raw material in this research. To make a better utilization of the plant oil, the fatty acid composition was firstly analyzed by methanolysis derivatization and GC–MS detection. The results in Table 1 show that the contents of two unsaturated acids, oleic acid (73.1%) and linoleic acid (23.8%), were considerably higher than other fatty acids with a total content of 3.1%.

**Table 1:** Fatty acid composition of *Amygdalus Pedunculata* plant oil

Fatty acid		wt%
Molecular formula	Name of fatty acid	
C16:0	Palmitic acid	2.0
C16:1	Palmitoleic acid	0.2
C18:0	Stearic acid	0.9
C18:1	Oleic acid	73.1
C18:2	Linoleic acid	23.8

If taking bio-aviation fuel as a target product, a traditional hydrodeoxygenation process, such as the UOP process, usually led to relatively higher hydrogen consumption because of the saturation of double bonds in unsaturated acids. At the same time, the hydrocracking process was usually unavoidable as the fatty acid in plant oil mostly contained 16 and 18 carbons, exceeding the requirements of bio-aviation fuel. Therefore, in order to get the utmost carbon utilization of the plant oil, a specific new process with lower hydrogen consumption and higher product yield needed to be developed, and according to the fatty acid composition characteristics of the plant oil, a newly designed process for transforming the plant oil into aviation fuel and α-olefin with lower hydrogen consumption and higher product yield was shown in Scheme 1.

Firstly, the plant oil was transformed into fatty acid methyl esters by an enzyme-catalyzed process previously reported by our group [29]. Secondly, the fatty acid methyl esters were subjected to a partial catalytic hydrotreatment to transform linoleic acid methyl ester into oleic acid methyl ester, since linoleic acid methyl ester present in the plant oil will lead to side reactions and the formation of undesired products in the olefin cross-metathesis reaction process [30]. It should be noted that the first and second step could be reversed according to the feedstock properties. Furthermore, the partial hydrogenation step could be omitted if the oleic acid content is sufficiently high in the starting oil. Then, the oleic acid methyl ester was reacted with ethylene catalyzed by a Grubbs’ catalyst used for olefin cross-metathesis. After the olefin cross-metathesis step, two products, α-olefin with high value and 1-decenoic acid methyl ester, ideal precursor for bio-aviation fuel production, were obtained. Finally, the 1-decenoic acid methyl ester was transformed into aviation fuel by catalytic hydrodeoxygenation and hydro-isomerization without a carbon losing hydrocracking process. As a result of the newly developed process, the plant oil was efficiently transformed into a bio-aviation fuel product and a value-added product, α-olefin. The detail results of catalyst and condition screening work are shown in “Catalytic conversion process of plant oil” section.

### Catalytic conversion process of plant oil

#### Partial catalytic hydrotreatment of the plant oil

**Fig. 1:**
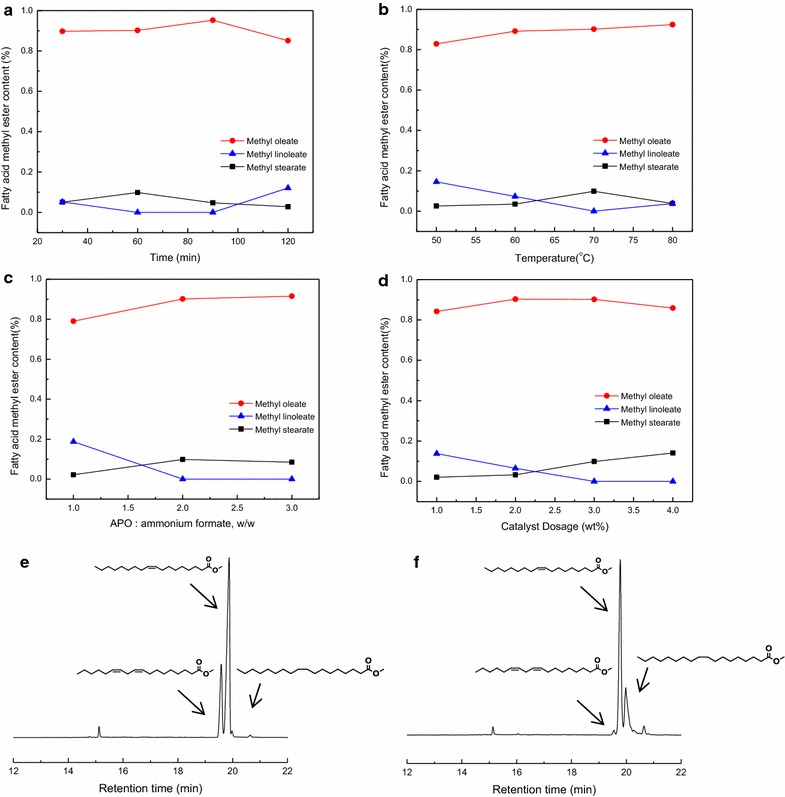
**a**–**d** Reaction condition screening of catalytic transfer hydrogenation; GC/MS chromatograms of the reactants and products after catalytic transfer hydrogenation: **e** reactants; **f** products after 90 min. Reaction conditions: **a** 70 °C, APO:ammonium formate = 1:2 (w/w), catalyst dosage 3% wt; **b** reaction time 60 min, APO:ammonium formate = 1:2 (w/w); catalyst dosage 3% wt; **c** 70 °C, reaction time 60 min, catalyst dosage 3% wt; **d** 70 °C, reaction time 60 min, catalyst dosage 3% wt

To avoid side reactions and the formation of undesired product in olefin cross-metathesis, the methyl *Amygdalus Pedunculata* plant oil (APO) was firstly pretreated by partial catalytic hydrotreatment to transform linoleic acid into oleic acid, using a Pd/C catalyst. To get higher selectivity, the reaction conditions, including reaction time, reaction temperature, weight ratio of two substrates, and catalyst dosage were optimized, and the results are shown in Fig. 1. Under the optimal conditions, being reaction time 90 min, reaction temperature 70 °C, APO:ammonium formate = 1:2 (w/w), and 3 wt% catalyst dosage, the content of oleic acid methyl ester (OAME) could reach up to 90.2%. It should be noted that the partial catalytic hydrotreatment of plant oil is more important when the starting oil has a high poly-unsaturated fatty acid content, although the partial catalytic hydrotreatment disclosed in this paper is also applicable to oil with high poly-unsaturated fatty acid content. For example, the partial hydrotreatment of an oil, rich in poly-unsaturated fatty acid, was shown in the additional information (Additional file 1: Table S1).

#### Olefin cross-metathesis between oleic acid methyl ester and ethylene

**Fig. 2:**
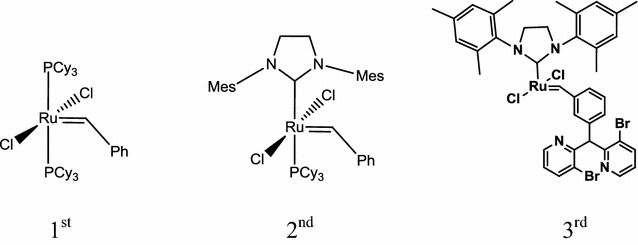
Chemical structure of the commercial Grubbs’ catalysts used in this research

The olefin cross-metathesis step was the key step of the whole process. To choose a catalyst suitable for olefin cross-metathesis between OAME and ethylene, three kinds of commercial Grubbs’ catalysts were accessed. Their chemical structures are shown in Fig. 2. The olefin cross-metathesis results between OAME and ethylene are shown in Table 2 indicating that the 1st Grubbs’ catalyst was most suitable for the olefin cross-metathesis. Although the 2nd and 3rd Grubbs’ catalysts showed high reaction activity in some cases, the ligand of ruthenium of these two catalysts does not lead to higher performance in the olefin cross-metathesis between oleic acid methyl ester and ethylene.

**Table 2:** Ethenolysis of methyl oleate using different catalysts

Catalyst	Conversion (%)	Selectivity (%)	Yield (%)
1st Grubbs’ catalyst	94.854	100	94.85
2nd Grubbs’ catalyst	97.044	55.37	53.73
3rd Grubbs’ catalyst	56.542	32.70	18.49

Reaction conditions: catalyst 0.3%, CH_2_=CH_2_, 40 °C, 120 min

**Fig. 3:**
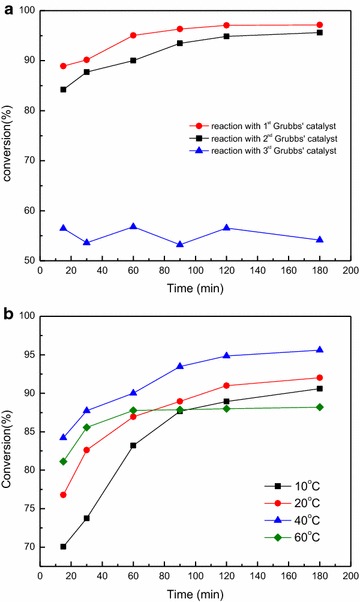
Metathesis catalyst screening and reaction condition optimization. **a** Effects of catalyst species on metathesis reaction conversion, **b** effects of reaction temperature and time on the metathesis reaction conversion

The influence of different reaction conditions was further investigated, and the results is shown in Fig. 3. With an increase in the reaction temperature, firstly, the conversion ratio was increased. However, when the reaction temperature reached 60 °C, the conversion decreased. This result indicated that there existed an optimal temperature (40 °C) in olefin cross-metathesis between oleic acid methyl ester and ethylene. As for the reaction time, the reaction curve almost reached its maximum at 120 min, and a further increase of the reaction time did not increase the conversion. Therefore, 120 min was chosen as optimal reaction time. Under optimal reaction conditions and with the 1st Grubbs’ catalyst, the conversion ratio, selectivity, and yield could reach up to 94.85, 100, and 94.85%, respectively.

#### Catalytic hydrodeoxygenation of 1-decenoic acid methyl ester

In order to transform 1-decenoic acid methyl ester into aviation fuel by catalytic hydrodeoxygenation and hydro-isomerization, Pt/ZSM-22 catalyst was synthesized and characterized according to our previously publication [31]. The characterization results for the catalyst were shown in Additional file 2: Figure S1. The acidic property of ZSM-22 determined by NH_3_-TPD and Py-FT-IR is shown in Additional file 2: Figure S1a, b. A major peak at high temperature, as shown in Additional file 2: Figure S1a, indicated that the Pt/ZSM-22 catalyst had a strong acidity. The incipient wetness impregnation method was used to introduce Pt into the supporting material. Due to the high dispersion of Pt on the bi-functional surface, no diffraction peak from Pt was found in Additional file 2: Figure S1c. According to the N_2_ adsorption–desorption isotherm results, shown in Additional file 2: Figure S1d, the calculated textural properties of the Pt/ZSM-22 catalysts were shown in Additional file 3: Table S2. The XPS spectra of Pt 4f for fresh and used Pt/ZSM-22 catalyst were shown in Additional file 2: Figure S1e, f, respectively. Furthermore, the Pt content on Pt/ZSM-22 catalyst was controlled to be 0.5% and confirmed by ICP analysis.

**Fig. 4:**
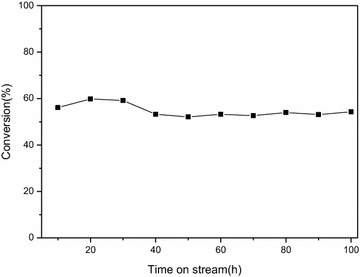
Catalyst (Pt/ZSM-22) stability of methyl decanoate hydrodeoxygenation process in a continuous flow reactor

The hydrodeoxygenation and hydro-isomerization reactions catalyzed by Pt/ZSM-22 catalyst were carried out in a high-pressure fixed-bed reactor. The reaction curve and durability of the catalyst are shown in Fig. 4. The conversion of 1-decenoic acid methyl ester to aviation fuel could be maintained around 60.0% for 100 h. Moreover, the XPS spectra shown in Additional file 2: Figure S1e, f also illustrated that the Pt in the catalyst retained its zero valence after reaction, which reflected the good stability of Pt/ZSM-22 catalyst.

### Economic and cost accounting of the newly designed process

Combining the olefin cross-metathesis results in “Olefin cross-metathesis between oleic acid methyl ester and ethylene” section and the results of the catalytic conversion process in “Catalytic hydrodeoxygenation of 1-decenoic acid methyl ester” section, the process planned in Scheme 1 was successfully achieved in this work. According to the results of the catalytic process in Scheme 1, a flow chart of the combination of olefin cross-metathesis and hydrotreatment process with handling capacity of 18 ton plant oil per hour was carried out by ASPEN software. The partial catalytic hydrotreatment, enzymatic catalyzed transesterification, olefin cross-metathesis and catalytic hydrodeoxygenation, and hydro-isomerization process were taken into consideration in the process simulation. According to the material balance results, for every ton plant oil input, the output of 1-decene and of bio-aviation fuel achieved was 0.42 and 0.32 ton, respectively. Without the partial hydrogenation process, the output of 1-decene dropped to 0.37 ton and the bio-aviation fuel yield was almost unchanged. When compared with former research and commercial bio-aviation producing processes e.g., UOP/Honeywell or NESTE [32], the carbon efficiency of this newly designed process was much higher than the reported. For example, according to the material balance, every ton of plant oil input yielded an output of 1-decene and of bio-aviation fuel as 0.42 and 0.32 ton, respectively. The products’ yield by weight could reach up to 75.9%, but the yield of former research and commercial bio-aviation producing process could only be around 35–45% [32]. Meanwhile, the carbon efficiency of this new process could be more than 90% and yielded a valuable chemical, α-olefin.

## Conclusions

In this research, we designed a highly efficient conversion process of plant oil into bio-aviation fuel and valuable chemicals by combination of enzymatic transesterification, olefin cross-metathesis, and hydrotreatment, with prepared and selected catalysts. The reaction conditions were optimized. Plant oil was transformed into bio-aviation fuel and a high value α-olefin product with high carbon utilization. Using the 1st Grubbs’ catalyst, the conversion, selectivity, and yield of OAME to α-olefin could reach up to 94.85, 100, and 94.85%, respectively. The yield of bio-aviation fuel could also reach up to 60% using Pt/ZSM-22 catalyst for hydrodeoxygenation and hydro-isomerization. Moreover, the carbon efficiency analysis of the newly designed process illustrated that the novel process was more economically feasible than the traditional hydrotreatment process.

## Methods

### Materials

Palladium on carbon, Pd/C (5 wt.% loading, matrix activated carbon support), was supplied by Aldrich. Ammonium formate (97%, water < 3%) was purchased from Alfa Aesar. Plant oil was extracted from *Amygdalus Pedunculata* seeds. The commercial 1st (CAS No. 172222-30-9), 2nd (CAS No. 246047-72-3), and 3rd (CAS No. 900169-53-1) generation Grubbs catalysts were purchased from Energy Chemical Co. Toluene, *n*-hexane, ethanol, methanol, ethene were all of analytical grade and purchased from Beijing Chemical Works Co.

### Preparation of Pt/ZSM-22 catalysts

Ten grams of ZSM-22 and 100 mL of ammonium nitrate (1 M) were added into a 250-mL three-neck flask, and the mixture was reacted at 80 °C for 2 h. The solid phase was then separated by vacuum filtration and twice dried in an oven at 100 °C for 2 h. The product was then subjected to a muffle furnace roast at 550 °C for 2 h. After these pre-treatment processes, the HZSM-22 was obtained. For Pt/ZSM-22 preparation, the wetness impregnation method was used to introduce Pt into the support. After wetness impregnation, the precursor of Pt/ZSM-22 was put into a tube furnace for hydrogenating at 420 °C in 4 h.

### Characterization methods

The organic products were analyzed by Agilent GC–MS (7890A, 5975C) with HP-5 column (30 m × 0.25 mm). The temperature program was started at 50 °C for 1 min, then ramped to 200 °C at a rate of 3 °C min^−1^ and held for 2 min.

X-ray powder diffraction (XRD) patterns of the catalyst were obtained using a Bruker diffractometer, and data were recorded in the 2 θ range of 5°–60° operated at 40 kV and 120 mA with Cu Kα radiation with an angular step size of 0.05° and a counting time of 8 s step^−1^.

Transmission electron microscopy (TEM) images were obtained using a FEI Tecnai G2 F30 twin microscope operated at 300 kV for high-resolution measurements. The samples were dispersed in ethanol, and deposited on a holey film on a lacey support film.

Low-temperature N_2_-adsorption was measured at − 196 °C using a Micromeritics ASAP 2020 HD 88 surface area and porosity analyzer to obtain the microporous and mesoporous porosities. The calcined samples were degassed at 350 °C under a vacuum of 1.33 × 10^−3^ Pa for 8 h and then switched to the analysis station for adsorption–desorption at liquid nitrogen temperature.

To characterize the acidity, the temperature-programmed desorption of ammonia (NH_3_-TPD) was carried out in a Dynamic Chemisorption Analyzer (Micromeritics Autochem 2910 II). Following in situ pre-treatment in flowing He (20 cm^3^ min^−1^) at 500 °C for 2 h, the samples were saturated with 10 vol% NH_3_ at 100 °C for 30 min. Physisorbed NH_3_ was removed by purging with He. The desorption of NH_3_ was monitored over the range of 100–700 °C at a heating rate of 10 °C min^−1^.

The FTIR spectra of pyridine adsorption were obtained on a Nicolet Magna-IR 750 spectrometer as KBr pellets. 10 mg of sample was pressed into a self-supporting wafer of about 10 mm in diameter. The wafer was first evacuated in situ in an IR cell at 450 °C for 2 h. Pyridine was then introduced into the cell at room temperature until the saturated adsorption was reached. Finally, the desorption of pyridine was stepwise performed at increasing temperatures under 1 × 10^−3^ Pa pressure and the spectra were recorded correspondingly.

The XPS analyses were carried out on a ESCALAB 250 spectrometer equipped with a Al-K_α_ source. Deconvolution of the XPS spectra was carried out using the software XPS Peak 4.1 [33].

The bulk chemical composition was analyzed by inductively coupled plasma-atomic emission spectrometer (ICP-AES, Thermo Fisher iCAP 6000 series).

### Catalytic reaction

#### Enzyme-catalyzed esterification of Amygdalus Pedunculata plant oil

Fifty grams of hydrogenated *Amygdalus Pedunculata* plant oil and 5 g of immobilized *Candida* sp. 99–125 lipase were added into a 500-mL three-neck flask. The system was reacted at 40 °C for 24 h. During the reaction time, 5.45 g of methanol was totally added into the flask for 3 times, 1.818 g for every 8 h [29]. After cooling to room temperature, the immobilized lipase was removed by filtration. Then, the filter liquor was transferred into a flask vacuum distillation to obtain the methyl ester products. The product was characterized by GC–MS.

#### Catalytic transfer hydrogenation of methyl *Amygdalus Pedunculata* plant oil

3 mol% of palladium on carbon catalyst with 25 g of methyl *Amygdalus Pedunculata* plant oil and 20 mL ethanol were added into a 100-mL well-dried flask at 70 °C. 3.60 g of ammonium formate was dissolved in 10 mL deionized water and transferred into the flask with constant pressure funnel in 5 min. The system reacted at 70 °C for 90 min. After the reaction mixture was cooled to room temperature, hexane was added into the mixture and transferred to a 30-mL centrifuge tube. After centrifugation, the liquid was transferred into a flask vacuum distillation to obtain the products. The products were characterized by GC–MS after esterification with methanol.

#### Olefin cross-metathesis reactions

Before the reaction, toluene was purified, dehydrated, and purged with N_2_ for 24 h. 5 g of vegetable oil methyl ester and 3 mol% of the first generation Grubbs’ catalyst were added in a well-dried flask containing 30 mL of super-dried toluene. Ethene was subsequently blown into the reaction system, stirred at 40 °C. The catalyst and the products were separated by flash chromatography. The products were characterized by GC–MS.

The conversion was calculated as the weight of methyl oleate in the products divided by the weight of methyl oleate in the feedstock, which means the transformed methyl oleate in the metathesis reaction. The selectivity was calculated as the total weight of methyl dec-9-enoate and 1-decene in liquid products divided by the theoretical total weight of dec-9-enoate and 1-decene from methyl oleate, which indicated the selectivity of the catalyst in the metathesis reaction. The yield was calculated by conversion time selectivity, which represented the catalyst efficiency of transforming methyl oleate and ethylene to desired methyl dec-9-enoate and 1-decene.

The formulas for the conversion and selectivity calculation are as follows:

$${\rm{Conversion}} = \left( {1 - \frac{{{\rm{weight \,of \,methyl\,oleate\,in\,products }}}}{{{\rm{weight\,of\,methyl\,oleate\,in\,feedstock}}}}} \right) \times 100\%$$

$${\text{Selectivity}} = \frac{{{\text{weight}}\;{\text{of}}\;{\text{methyl}}\;{\text{dec-}}9{\text{-enoate}}\;{\text{in}}\;{\text{products}} + {\text{weight}}\;{\text{of}}\;1{\text{-decene}}\;{\text{in}}\;{\text{products}}}}{{{\text{theoretical}}\;{\text{weight}}\;{\text{of}}\;{\text{methyl}}\;{\text{dec-}}9{\text{- enoate}} + {\text{theoretical}}\;{\text{weight}}\;{\text{of}}\;{\text{1-decene}}}} \times 100\% .$$

#### Catalytic hydrodeoxygenation of methyl decanoate

Five grams of prepared Pt/ZSM-22 was fed into a fixed-bed reactor. N_2_ purging was used to remove the air in the fixed-bed reactor during 30 min. The system pressure and the H_2_ flow rate at the outlet were thereafter controlled by a backup pressure valve, at 3 MPa and 100 mL min^−1^. After the temperature was increased to 340 °C, the reactant was pumped into the reactor at a rate of 0.2 mL min^−1^. A condenser at – 20 °C was employed for collecting products, and the reaction mixture was sampled every hour for analysis by GC–MS.

#### Materials balance calculation

The mass balance of the overall process was calculated based on ASPEN v8.6. A flow sheet as scheme 1 was developed. The input data used were from “Catalytic conversion process of plant oil” section.

## Additional file

**Additional file 1: Table S1.** Fatty acid profile of poly-unsaturated fatty acid rich oil before and after partial hydrogenation.
**Additional file 2: Fig. S1.** Characterization of Pt/ZSM-22 catalyst. (a) NH3-TPD profile of ZSM-22; (b) FTIR spectrum of pyridine adsorption on ZSM-22; (c) X-ray pattern of Pt/ZSM-22; (d) nitrogen adsorption–desorption isotherms of Pt/ZSM-22; XPS spectra of Pt 4f for (e) Pt/ZSM-22 before reaction and (f) Pt/ZSM-22 after reaction.
**Additional file 3: Table S2.** Textural properties of the Pt/ZSM-22 catalysts.
