# Supplementary material for: Highly efficient conversion of plant oil to bio-aviation fuel and valuable chemicals by combination of enzymatic transesterification, olefin cross-metathesis, and hydrotreating
Source: Biotechnol Biofuels. 2018 Feb 7;11:30. doi: 10.1186/s13068-018-1020-4 (PMC5801801; doi:10.1186/s13068-018-1020-4)
Supplement: Supplementary file 1 — Additional file 1: Table S1. Fatty acid profile of poly-unsaturated fatty acid rich oil before and after partial hydrogenation. [file 13068_2018_1020_MOESM1_ESM.docx]

Table S1 Fatty acid profile of poly-unsaturated fatty acid rich oil before and after partial hydrogenation

| Fatty acid profile | 16:0 | 16:1 | 18:0 | 18:1 | 18:2 | 18:3 | 20:0 | 20:1 | Others |
| --- | --- | --- | --- | --- | --- | --- | --- | --- | --- |
| Original/% | 36.2 | 2.87 | 3.3 | 24.5 | 19.2 | 11.1 | 0.71 | 1.11 | 1.01 |
| After hydrogenation/% | 35.8 | 3.1 | 6.8 | 51.87 | 0.30 | 0.17 | 0.57 | 1.31 | 0.08 |
