# Supplementary material for: Highly efficient conversion of plant oil to bio-aviation fuel and valuable chemicals by combination of enzymatic transesterification, olefin cross-metathesis, and hydrotreating
Source: Biotechnol Biofuels. 2018 Feb 7;11:30. doi: 10.1186/s13068-018-1020-4 (PMC5801801; doi:10.1186/s13068-018-1020-4)
Supplement: Supplementary file 2 — Additional file 2: Fig. S1. Characterization of Pt/ZSM-22 catalyst. (a) NH3-TPD profile of ZSM-22; (b) FTIR spectrum of pyridine adsorption on ZSM-22; (c) X-ray pattern of Pt/ZSM-22; (d) nitrogen adsorption–desorption isotherms of Pt/ZSM-22; XPS spectra of Pt 4f for (e) Pt/ZSM-22 before reaction and (f) Pt/ZSM-22 after reaction. [file 13068_2018_1020_MOESM2_ESM.docx]

**Supporting Information**

**Highly Efficient Conversion of Plant Oil to Bio-aviation fuel and Valuable Chemicals by Combination of Enzymatic Transesterification, Olefin Cross-metathesis and Hydro-treating**

Meng Wang ^a^, Mojin Chen ^a, b^, Yunming Fang ^a, b^[[1]](#footnote-1)^*^, Tianwei Tan ^a^

^a^ ^[[2]](#footnote-2)^National Energy Research Center for Biorefinery, College of Life Science and Technology, Beijing University of Chemical Technology, Beijing, 100029, PR China.

^b^ Department of Chemical Engineering, Beijing University of Chemical Technology, Beijing, 100029, PR China.

a b

c d

e f

Fig. S1 Characterization of Pt/ZSM-22 catalyst

(a) NH_3_-TPD profile of ZSM-22; (b) FTIR spectrum of pyridine adsorption on ZSM-22; (c) X-ray pattern of Pt/ZSM-22; (d) nitrogen adsorption–desorption isotherms of Pt/ZSM-22; XPS spectra of Pt 4f for (e) Pt/ZSM-22 before reaction and (f) Pt/ZSM-22 after reaction

1. Corresponding author.

   E-mail: fangym@mail.buct.edu.cn(Y. Fang) [↑](#footnote-ref-1)
2. [↑](#footnote-ref-2)
