# Supplementary material for: Highly efficient conversion of plant oil to bio-aviation fuel and valuable chemicals by combination of enzymatic transesterification, olefin cross-metathesis, and hydrotreating
Source: Biotechnol Biofuels. 2018 Feb 7;11:30. doi: 10.1186/s13068-018-1020-4 (PMC5801801; doi:10.1186/s13068-018-1020-4)
Supplement: Supplementary file 3 — Additional file 3: Table S2. Textural properties of the Pt/ZSM-22 catalysts. [file 13068_2018_1020_MOESM3_ESM.docx]

Table S2 Textural properties of the Pt/ZSM-22 catalysts

| Material | Surface area(m^2^∙g^-1^) | Pore volume(cm^3^∙g^-1^) | Pore diameter(nm) |
| --- | --- | --- | --- |
| Pt/ZSM-22 | 210.02 | 0.34 | 6.48 |
